# Epidemic Cerebrospinal Meningitis

**Published:** 1899-02-11

**Authors:** 


### EPIDEMIC CEREBROSPINAL MENINGITIS.

THE paper read by Dr. Bruce Low on Epidemic Cerebrospinal Meningitis at the last meeting of the Epidemiological Society serves to remind us of a disease in which, in consequence of its presumed infrequency, but little interest has been taken by the mass of the profession. It seems not improbable, however, that it is a good deal more common than it has been imagined to be; and Dr. Low suggests that many deaths ascribed to influenza are probably due to this malady, while no doubt some of the alleged instances of recovery from tuberculous meningitis are due to confusion between the two diseases. Overcrowding and cold and wet, either directly or as leading to neglect of ventilation, seem to be predisposing causes of the epidemic form, but there can now be no doubt that the actual occurrence of the disease is due to an infection. Many attempts have been made to isolate the microbe on which this infection depends; but, although micrococci were discovered in the cerebrospinal fluid, both of epidemic and other forms of meningitis, they were supposed to be identical with the pneumococcus of Fraenkel. Weichselbaum, in 1887, described a peculiar form of micrococcus, to which he gave the name of diplococcus intracellularis; but it was not until 1895 that Jaeger proved that this organism, which he called tetracoccus, was the cause of the epidemic cerebrospinal fever, and that its presence distinguished this from some other forms of meningitis. His conclusions have since been fully confirmed by numerous observers in Germany, Austria, and America, and by Dr. Still in this country. The discovery of this micro-organism, however, and of its causative relation to the disease in question, has also had the effect of somewhat widening our conception of its relations and varieties, for it would appear that the same organism is also the cause of infantile paralysis and of the posterior basal meningitis of infants. The word "epidemic" as forming part of the descriptive title of the disease would then seem to be somewhat out of place, for it may occur in a purely sporadic manner. Four forms of the disease are usually recognised—the typical, the purpurous, the fulminant, and the abortive or chronic. Many cases do not accord with the definitions given in the books of the typical disease; and, indeed, in view of the fact that influenza may render the individual specially susceptible to infection with the pneumococcus, and that when this organism has gained access it may itself set up a meningitis, and thus simulate the symptoms of the specific disease, it would seem impossible in some cases to arrive at a certain diagnosis were it not for the fact that by lumbar puncture cerebrospinal fluid can be obtained for examination when the tetracoccus may be discovered. Dr. Low pointed out that lumbar puncture, if performed below the end of the cord, is devoid of danger, easy, and almost painless, while in the present state of our knowledge it is almost indispensable to correct diagnosis in all but the typical cases. Now it is just these links which we want to trace. The fact that animals are subject to the same disease raises many important questions as to the manner in which the tetracoccus maintains its extra corporeal existence.

